# Clinical characteristics associated with the prescribing of SSRI medication in adolescents with major unipolar depression

**DOI:** 10.1007/s00787-016-0849-y

**Published:** 2016-04-28

**Authors:** Lesley Cousins, Kirstie J. Whitaker, Barry Widmer, Nick Midgley, Sarah Byford, Bernadka Dubicka, Raphael Kelvin, Shirley Reynolds, Christopher Roberts, Fiona Holland, Barbara Barrett, Robert Senior, Paul Wilkinson, Mary Target, Peter Fonagy, Ian M Goodyer

**Affiliations:** 1 Developmental Psychiatry Section, University of Cambridge, Douglas House, 18b Trumpington Road, Cambridge, CB2 8AH UK; 2 Anna Freud Centre, University College London, London, UK; 3 Centre for the Economics of Mental and Physical Health, Kings College London, London, UK; 4 Institute of Brain and Behaviour and Mental Health, University of Manchester, Manchester, UK; 5 School of Medicine, University of East Anglia, Norwich, UK; 6 Biostatistics Group, Institute of Population Health, University of Manchester, Manchester, UK; 7 The Tavistock and Portman NHS Foundation Trust, Tavistock Centre, London, UK; 8 Research Department of Clinical, Educational and Health Psychology, University College London, London, UK; 9 Cambridgeshire and Peterborough NHS Foundation Trust, Cambridge, UK; 10 Lancashire Care Foundation Trust, Preston, UK

**Keywords:** Adolescents, Depression, Antidepressants, SSRIs, Risk, Self-harm

## Abstract

Unipolar major depressions (MD) emerge markedly during adolescence. National Institute for Health and Care Excellence (NICE) UK recommends psychological therapies, with accompanying selective serotonin reuptake inhibitors (SSRIs) prescribed in severe cases only. Here, we seek to determine the extent and rationale of SSRI prescribing in adolescent MD before entering a randomised clinical trial. SSRI prescribing, together with their clinical characteristics was determined in 465 adolescent patients with MD prior to receiving a standardised psychological therapy as part of the Improving mood with psychoanalytic and cognitive therapies (IMPACT) clinical trial. Overall, 88 (19 %) had been prescribed antidepressants prior to psychological treatment. The clinical correlates varied by gender: respectively, depression severity in boys and self-harming behaviours in girls. Prescribing also differed between clinical research centres. Medical practitioners consider severity of depression in boys as an indicator for antidepressant prescribing. Self-injury in girls appears to be utilised as a prescribing aid which is inconsistent with past and current revised UK NICE guidelines.

**Electronic supplementary material:**

The online version of this article (doi:10.1007/s00787-016-0849-y) contains supplementary material, which is available to authorized users.

## Introduction

MD is a significant health problem affecting a substantial proportion of the adolescent population worldwide [1]. The estimated 12 month period prevalence of MD in teenagers is 7.5 % affecting around twice as many girls as boys and around 1 in 4 of these will have a severe clinically referable condition [2, 3]. The long term consequences of adolescent emergent MD can include suicide, anxiety disorders, substance misuse and failure to both achieve educationally and in the work place [4]. These negative outcomes come at great economic cost to the UK and other national economies and therefore, ensuring that we manage MD effectively in this age group should be a priority for public mental health policy and practice.

The UK NICE guidelines are one of the few focused and standardised recommendations available for the management of adolescent MD within Europe and indeed worldwide. These standards have recently undergone revision. Previous guidance promoted the use of evidence-based psychological therapies as first line treatment, with SSRIs antidepressants constituting the pharmacological treatment of choice only if psychological therapies have first been tried without response, and only then, in combination with psychological therapy. The 2015 amendment to these guidelines allow combined SSRI and psychological therapy first line but continue to warn against the use of SSRIs on their own [5]. Both state that depression should be moderate to severe to warrant SSRI prescribing. Given that this guidance has recently been reviewed, we determined whether the current management of adolescent MD adheres to these guidelines, either revised or in their original form.

IMPACT is a pragmatic, multicentre randomised controlled trial of adolescents with MD [6]. It is designed to investigate the effectiveness of three different psychological interventions (short term psychoanalytic psychotherapy, cognitive behavioural therapy and manualised specialist clinical care) in reducing the risk of symptomatic depressive relapse in the medium term 12–18 months after beginning treatment. A clinical audit of NHS notes documenting prior treatments and medication prescribing in a sub-sample (n = 80) of participants prior to randomisation revealed that a marked proportion of audited cases had already been prescribed SSRI antidepressants prior to receiving their psychological intervention.

Given this apparent deviation in adherence to NICE guidelines, we wished to investigate the clinical rationale underlying this pre-trial prescribing of antidepressants across the whole of the trial cohort.

Our investigations were underpinned by four principle hypotheses; firstly, we hypothesised that pre randomisation prescribing would be based on severity or chronicity of clinical presentation. Secondly, we considered whether prescribing was based on perceived functional impairment or subjective quality of life rather than clinical signs and symptoms. Thirdly, we hypothesised that prescribing would be associated with overt risk behaviours such as self-harm and antisocial behaviour. We reasoned that practitioners may perceive such hazardous behaviours to be proxy indices of depression severity or consider such behaviours as requiring rapid pharmacological management. Finally, given previous findings of the importance of site-specific effects within RCTs, we investigated the importance of the research site individuals derived from, both in terms of SSRI prescribing and sample characteristics.

## Methods

### Improving mood with psychoanalytic and cognitive therapies (IMPACT) participants

IMPACT is a pragmatic randomised control trial which aims to determine if one of two specialist treatments, cognitive behavioural therapy (CBT) or short term psychoanalytic psychotherapy (STPP) are superior to  a reference practice-based brief psychosocial intervention (BPI), at preventing recurrence of clinically meaningful levels of depressive symptoms indicating potential relapse in adolescents who enter the trial with MD [6].

The study was conducted over three regions within the UK, East Anglia, North London and the North West of England incorporating 16 routine Child and Adolescent Mental Health (CAMH) clinics. Individuals were recruited to the study with moderate to severe depression and aged between 11 and 18 years. Exclusion criteria included generalised learning difficulties or a pervasive developmental disorder, pregnancy, currently taking another medication that may interact with an SSRI and being unable to stop this medication, substance misuse, primary diagnosis of Bipolar Type I disorder, schizophrenia or eating disorder. Of the 561 individuals who were referred and had baseline assessments, 470 were considered eligible with 5 later withdrawing consent, resulting in the recruitment of 465 overall. As part of the screening process prior to enrolment, individuals were asked if their current depressive illness was a first episode or a relapse. At this point, demographic information including their ethnicity was collected. Ethical approval was by the Cambridgeshire 2 research Ethics Committee, Addenbrooke’s Hospital Cambridge, UK and is therefore, in accordance with the ethical standards laid down in the 1964 Declaration of Helsinki and its later amendments. Follow up was undertaken with repeated reassessments at up to 86 weeks after randomisation to evaluate recurrence of clinical level depressive symptoms. The further characterisation of the sample will be available with the publication of the IMPACT trial during 2016 [7].

## Assessment instruments

### SSRI prescribing

Antidepressant prescribing prior to entering the study was determined using the Child and Adolescent Service Use Schedule (CA-SUS) [8]. The CA-SUS is an interview-based measure to collect data on service use and was designed for use within mental health populations, including young people with depression [8]. As well as ascertaining the current prescribing of psychotropic medication, it includes information on the individual’s accommodation, education, use of hospital and community based health and social services, and any criminal activity or criminal justice sector contacts. Baseline prescribing information was available for 457 individuals of the 465 recruited to the study (98 %). Subsequent analyses presented here excluded participants for whom this information is not available.

## Interview measures

### Schedule for affective disorders and schizophrenia for school aged children, present and lifetime version (K-SADS-PL) [9]

We hypothesised that SSRI prescribing may be associated with depression severity, psychiatric comorbidity or chronicity. This was investigated using the K-SADS-PL. With regard to depressive symptoms, this refers to the 22 items used to establish the diagnosis of current depression in the K-SADS-PL. A score of 3 or ‘threshold’ within the K-SADS-PL was used to define a symptom being present. The comorbid diagnoses considered, according to DSM-IV criteria, were panic with and without agoraphobia, separation anxiety, avoidant disorder, specific phobias, general anxiety disorder, obsessive compulsive disorder, post-traumatic stress disorder, anorexia nervosa, bulimia nervosa, attention deficit disorder, oppositional defiant disorder, conduct disorder, alcohol abuse and substance and alcohol dependence. Psychotic symptoms were also recorded but individuals with a diagnosis of mania or schizophrenia were excluded from the study. These data were available for all the trial participants. Using the K-SADS-PL, the age of onset of depressive illness was also compared between groups [data available for 282 individuals (62 %)]. The K-SADS-PL has been shown in numerous studies to have high inter-rater reliability and construct validity [9].

## Self-report measures

### Moods and feelings questionnaire (MFQ) [10]

The MFQ was also used to investigate if depression severity was associated with SSRI prescribing prior to randomisation in the IMPACT study. It is a 33 item self-report measure of depressive symptoms consisting of a list of descriptive phrases with subjects being asked to rate their symptoms over the past 2 weeks. Re-test reliability and criterion validity are reported to be high [11]. Cronbach’s alpha within our study was 0.92. Items are scored from 0 to 2 (never = 0, sometimes = 1, mostly or always = 2), yielding a maximum score of 66. These data were available for 462 out of the 465 study participants (99 %).

### Health of the nation outcome scales for children and adolescents (HONOSCA) [12]

The HONOSCA is a measure of a young person’s total mental health problems. It measures 15 items including psychiatric symptoms, behaviours, family, social and school functioning. Each item is scored from 0 to 4 where 0 indicates no problems and 4 very severe problems. It is known to be reliable, sensitive to change and correlates well with the clinician’s judgement of the young person’s outcome [13]. HONOSCA data were available for 432 of the study participants (93 %).

### Euroqol 5 dimension (EQ-5D) questionnaire [14]

The EQ-5D was used to investigate whether SSRI prescribing prior to randomisation in the IMPACT study was associated with functional impairment or quality of life. It is a standardised measurement of health status and health-related quality of life. The EQ-5D-3L version was used which consists of five dimensions (mobility, self-care, usual activities, pain/discomfort and anxiety/depression), recorded on one of three levels (no problems = 1, some problems = 2, extreme problems = 3). This allowed individuals to be classified into one of 243 health states. For example, 11,111 representing no problems in any dimension and 33333 representing extreme problems in all five dimensions. A summary index value was derived by applying general population weights to each health state [15]. The summary scores range from 1 (perfect health) to 0 (death) with negative scores indicating states considered to be worse than death. The EQ-5D was available for 436 of the individuals (94 %).

### Modified risk taking and self-harming inventory for adolescents (RTSHIA) [16]

The RTSHIA, a self-report instrument for assessing life-time self-harm and risk taking behaviour in adolescents, was used to investigate whether SSRIs are prescribed to adolescents based on hazardous or risk-taking behaviour. The version used differs from the original in that 3 questions were omitted regarding risky sexual behaviour. The version used contained 31 items answered on a Likert scale (3 = many times, 2 = more than once, 1 = once, 0 = never) and scored as a total for hazardous behaviour ranging from 0–93. Subscales for risk taking and self-harming were examined as well as the total score. Examples of questions that related to risk taking but not self-harm included drug and alcohol use, staying out late without parental knowledge and actively placing oneself in risky situations such as cheating at school and shop lifting. Cronbach alpha scores for each component of the RTSHIA within our study were; total = 0.90, risk taking = 0.80, self-harming = 0.90. This was available for 436 individuals of those entered into the trial (94 %).

### Behavioural checklist [17]

We also considered if antisocial behaviour may be considered a hazardous behaviour associated with prescribing. This was investigated using the Behavioural Checklist, an 11-item screen for current symptoms of antisocial behaviour derived from DSM-IV criteria and converted to a self-report format. It is scored on a 4 point Likert scale (always = 3, mostly = 2, sometimes = 1, never = 0) with a range of 0–44. Within our study, Cronbach alpha for the Behavioural Checklist was 0.79. The Behavioural Checklist was completed by 450 of the study participants (97 %).

## Statistical analysis

Many of the measures we collected were found to be non-normally distributed. Therefore, statistical tests between two groups were completed using the Wilcoxen Mann–Whitney test. The Chi-squared statistic was used to compare groups based on gender, research centre and ethnicity. To address the issue of missing data, scores for the MFQ, behavioural checklist and RTSHIA total and sub-scores were pro-rated.

Comparison of prescribing between research centres was also undertaken using logistic regression. A multinomial logistic regression analysis was undertaken to investigate the potential effects of research centre on the population characteristics with regard to gender, quality of life (EQ5D), HONOSCA score, self-harm, depression severity (as measured by MFQ), age and ethnicity. The relationship between SSRI prescribing and the predictors mentioned above, including research centre, was also tested using logistic regression analysis. Prior to this, we undertook a logistic regression using gender as a predictor to ascertain if there were gender effects on the parameters measured. Based on this, the model was split by gender to establish if there were differences in the predictors for prescribing between males and females.

Data were analysed using STATA Version 13 [18] for Windows PC.

## Results

### Demographics

Data regarding antidepressant prescribing prior to randomisation were available for 457 out of the 465 individuals recruited to the IMPACT trial. Of these, 89 (19.5 %) had been prescribed an antidepressant before entering the trial. The only antidepressants that had been prescribed were SSRIs with the majority (83 %) taking fluoxetine, as per NICE guidelines. The remaining individuals were prescribed citalopram (10 %) or sertraline (6 %). One individual was documented as taking an SSRI but the type of SSRI was not reported.

Prior to entering the study, individuals were asked if this was their first episode of depression. This information was available for 396 (85 %) of the study’s participants. There was no difference in any of the demographic variables (age, gender, ethnicity, MFQ score, age of onset of depression, number of depressive symptoms and number of comorbid disorders) measured between those for whom this information was available and those for whom it was not. Of those with available information, 370 (93.4 %) reported their current depressive illness as a first episode, the rest reporting a recurrence. Of the 77 individuals prescribed antidepressants for whom screening information was available, 73 reported their current depressive illness as being the first episode (94.8 %).

As shown in Table 1, individuals did not differ with regard to age, gender or ethnicity (white vs non-white) regardless of whether or not they were prescribed SSRIs. There was, however, a main effect of treatment centre, which will be discussed in more detail below.

**Table 1:** Baseline demographic for individuals prescribed SSRI antidepressants compared with those who were not

	No SSRI prescribed (*N* = 368)		SSRI prescribed (*N* = 89)		Statistic	*p*
	Median	IQR	Median	IQR		
Age (years)	15.6	14.6–16.8	15.7	15.0–16.8	*z* = 1.1	0.27
Age of onset of depression^a^	14.0	12.0–15.0	14.0	13.0–15.0	*z* = 0.1	0.89

	*n*	%	*n*	%		
Research centre						
East Anglia	126	34.2	54	60.6	*χ* ^2^ = 21.3	<0.000
London	112	30.4	14	15.7		
North West	130	35.3	21	23.6		
Gender						
Boys	89	24.2	27	30.3	*χ* ^2^ = 1.1	0.29
Ethnicity						
White	281	76.4	74	83.1	*χ* ^2^ = 1.4	0.23

^a^Derived from K-SADS-PL—schedule for affective disorders and schizophrenia for school aged children present and lifetime

### Illness severity

The severity of the young person’s current depressive illness was compared between those who had been prescribed antidepressants before entering the IMPACT study and those who had not been using both observer and self-rated measures (Table 2).

**Table 2:** Comparison of depression severity, functional impairment, hazardous behaviour and antisocial behaviour scores between individuals prescribed SSRIs and those who were not

	No SSRI prescribed (*N* = 368)		SSRI prescribed (*N* = 89)		Mann–Whitney *U* (*z* score)	*p*	Effect size (Cohen’s *d*)
	Median	IQR	Median	IQR			
MFQ score	46.0	38.2–54.0	50.0	42.0–55.7	1.8	0.072	
No of depressive symptoms^a^	8.0	7.0–10.0	8.0	7.0–11.0	0.6	0.53	
No of comorbid disorders^a^	1.0	0.0–2.0	1.0	0.0–2.0	0.6	0.54	
EQ5D	0.7	0.4–0.8	0.4	0.3–0.7	2.6	0.008	−0.32
HONOSCA	18.0	14.0–22.0	20.0	16.1–24.0	2.3	0.024	0.24
RTSHIA hazardous behaviour total	18.0	9.0–29.0	20.0	11.8–33.2	2.0	0.043	0.25
RTSHIA—risk taking	5.0	2.0–9.0	5.0	1.0–11.2	0.05	0.96	
RTSHIA—self-harm	12.0	5.0–21.0	15.5	7.0–24.2	2.19	0.028	0.28
Antisocial behaviour	3.0	1.0–5.0	2.0	0.0–4.0	2.3	0.021	−0.31

^a^Derived from K-SADS-PL—schedule for affective disorders and schizophrenia for school aged children present and lifetime

No significant difference between groups was observed in self-reported depression scores, the number of interview reported depressive symptoms (K-SADS PL), or the number of comorbid disorders associated with their depressive illness. There was also no difference in the age of onset of depressive illness obtained from the K-SADS PL.

### Quality of life/functional impairment

Individuals who had been prescribed an SSRI reported a lower level of health-related quality of life than those who were not taking antidepressants, as measured using the EQ5D (Table 2). They also reported higher HONOSCA scores, suggesting an overall poorer mental health status (Table 2). Together, these findings suggest that individuals who had been prescribed SSRIs subjectively perceived themselves as more functionally impaired.

### Hazardous behaviour

Individuals who had been prescribed antidepressants had higher self-reported lifetime histories of hazardous behaviour overall, as measured using the RTSHIA (Table 2).

When analysis of hazardous behaviour was split into risk taking and self-harm components, there was no difference between the groups with regard to risk taking behaviour, however, those who had been prescribed antidepressants had higher lifetime histories of self-harming behaviours.

### Antisocial behaviour

This hazard-related prescribing led us to the question of whether other forms of risky but non-depressive behaviour were also associated with antidepressant prescribing (Table 2). Surprisingly, individuals who had been prescribed antidepressants before entering the IMPACT study had fewer symptoms of self-reported antisocial behaviour compared with those who had not been prescribed antidepressant medication. It should be noted, however, that the median scores for each group are low in both, given the scale range (0–33).

### Prescribing differences across research centres

Prescribing rates of SSRI antidepressants differed significantly across the three UK research centres from which they were recruited (Table 1). Descriptively, 30 % of individuals recruited from East Anglia had been prescribed SSRIs prior to entering the study, compared with 11.1 and 13.9 % from London and the North West of England, respectively. Participants in East Anglia were, therefore, 2.7 (*z* = 3.4, *p* = 0.001) times more likely to be prescribed an SSRI compared with those recruited from the other two research centres.

These prescribing differences, however, did not explain the observed relationship between medication prescribing, quality of life/functional impairment and risky behaviour (Table 3). The pattern of quality of life, self-harming and antisocial behaviour scores were the same across research centres with the group who had been prescribed SSRIs prior to entering the study exhibiting lower EQ-5D scores (poorer health-related quality of life), higher HONOSCA scores (poorer overall mental health outcomes), higher self-harm/suicidality levels and lower antisocial behaviour levels, compared with those who had not been prescribed antidepressant medication.

**Table 3:** Relationship between SSRI prescribing, EQ5D, HONOSCA, hazardous behaviour, self-harm and antisocial behaviour across research centres

	East Anglia		London		North West	
	No SSRI mean (SD)	SSRI mean (SD)	No SSRI mean (SD)	SSRI mean (SD)	No SSRI mean (SD)	SSRI mean (SD)
EQ5D	0.59 (0.25)	0.48 (0.25)	0.56 (0.30)	0.48 (0.30)	0.59 (0.27)	0.53 (0.26)
HONOSCA	18.9 (5.2)	19.9 (5.6)	18.8 (6.5)	22.9 (6.6)	17.1 (6.4)	17.2 (5.7)
RTSHIA—self-harm	17.1 (10.8)	17.5 (11.1)	13.1 (9.8)	15.5 (10.6)	11.2 (9.7)	16 (13.7)
Antisocial behaviour	3.5 (3.2)	2.2 (2.3)	3.8 (3.3)	3.4 (3.3)	3.2 (3.4)	2.6 (2.7)

We tested for potential effects of centre on sample characteristics using a multinomial logistic regression (Supplementary Table 1). This showed that individuals recruited from London and the North West displayed lower self-harm scores compared with those recruited from East Anglia [median score (IQR): East Anglia: 16.0 (9.0–26.0), London: 13.0 (5.0–21.0), North West: 10.0 (4.0–20.5)]. Individuals from London were also slightly older [median age (IQR): East Anglia: 15.5 (14.7–16.3) years, London: 16.1 (14.8–17.2) years, North West: 15.6 (14.6–16.7) years] and more likely to be of a non-Caucasian ethnicity (East Anglia: 90 % Caucasian, London: 53 % Caucasian, North West: 84 % Caucasian). Adolescents from the North West had significantly lower HONOSCA scores compared with the East Anglian population [median score (IQR): East Anglia 19.2 (16.0–24.9), London: 20.0 (14.4–25.8), North West: 17.0 (13.0–21.3)].

### Gender differences in SSRI prescribing predictors

A logistic regression using gender as an interaction term allowed for any potential effects of gender on each of the univariates predicting SSRI prescribing to be investigated (Supplementary Table 2). Depression severity (as measured by MFQ) was added to the model as the difference between the two groups approached significance. Age was also added to the model in case this was shown to be a prescribing predictor when other interactions were controlled for. This showed that SSRI prescribing appeared to be more likely in girls with higher self-harm scores and boys with greater depressive symptoms (as measured by MFQ). This was confirmed by a further logistic regression analysis examining for main effects and interactions between SSRI prescribing and the possible predictors; quality of life, total mental health problems (as measured by the HONOSCA), self-harm, antisocial behaviour, depression severity, research centre and ethnicity. Given the findings from our first model, this analysis was undertaken for boys and girls separately and the results are presented in Table 4. They confirmed that the main predictor for prescribing differed based on gender. For girls, prescribing was associated with a history of self-harming behaviour. In contrast, prescribing for boys was associated with depression severity at randomisation. The area boys were recruited from also appeared to play a role as to whether they were prescribed SSRIs or not, they were more likely to be prescribed antidepressants if they came from East Anglia as opposed to London or the North West of the UK.

**Table 4:** Logistic regression analysis, demonstrating the relationship between quality of life, self-harm, antisocial behaviour and centre as predictors for SSRI prescribing at baseline

	Odds ratio	S.E.	z	p	95 % C.I.
Girls					
EQ5D—quality of life	0.50	0.63	−1.10	0.27	−1.92–0.54
HONOSCA—total mental health problems	1.05	0.03	1.57	0.12	−0.01–0.11
RTSHIA—self-harm	**1.03**	**0.02**	**2.00**	**0.045**	**0.001–0.065**
Behavioural checklist—antisocial behaviour	0.89	0.06	−1.78	0.076	−0.24–0.01
MFQ—depression severity	0.99	0.02	−0.71	0.48	−0.052–0.024
Age	1.17	0.13	1.19	0.23	−0.10–0.41
Research Centre: London^a^	0.44	0.46	−1.77	0.076	−1.70–0.09
Research Centre: North West^a^	0.61	0.37	−1.35	0.18	−1.23–0.23
Ethnicity	1.25	0.45	0.50	0.61	−0.65–1.10
Boys					
EQ5D—quality of life	0.64	1.10	−0.41	0.68	−2.60–1.71
HONOSCA—total mental health problems	1.00	0.05	0.10	0.92	−0.09–0.10
RTSHIA—self-harm	0.95	0.03	−1.39	0.17	−0.12–0.02
Behavioural checklist—antisocial behaviour	0.86	0.09	−1.67	0.095	−0.34–0.03
MFQ—depression severity	**1.07**	**0.03**	**2.04**	**0.042**	**0.002–0.13**
Age	1.32	0.18	1.53	0.13	−0.08–0.63
Research Centre: London^a^	**0.12**	**0.86**	**−2.52**	**0.012**	**−3.84** to **−0.48**
Research Centre: North West^a^	**0.15**	**0.66**	**−2.91**	**0.004**	**−3.22** to **−0.63**
Ethnicity	0.78	0.75	−0.34	0.74	−1.72–1.08

^a^Compared with East Anglia

## Discussion

We set out to explore the clinical characteristics that may account for the prescribing of antidepressants to adolescents with moderate to severe depression prior to entering the IMPACT study. To do this, we studied the baseline clinical characteristics of individuals including the severity/chronicity of depression, quality of life/functional impairment, self-harming/suicidality and other risk related antisocial behaviours.

The majority of individuals (>90 %) were experiencing their first episode of depression and did not report at recruitment receiving any formal psychological treatment before entering the trial. Therefore, at least 19 % of the study population received an SSRI without a concurrent psychological treatment.

So what are the clinical characteristics underlying the prior antidepressant prescribing in those adolescents with MD recruited subsequently to the trial? Within the total population, significant differences were noted in non-depressive symptoms and behaviours: those who had been prescribed SSRIs prior to entering the IMPACT study reported a reduced quality of life, higher subjective total mental health problems and higher levels of self-harm compared with those not prescribed. Unexpectedly, young people who had been prescribed SSRIs also reported lower antisocial behaviour symptoms. Perhaps, low antisocial behaviour in a presumed depressed adolescent is a contributory factor to SSRI prescribing. However, it should be noted that scores were low across the cohort, making interpretation of this result unclear.

We also identified significant differences between SSRI prescribing and the area within the UK the young person came from. Individuals from East Anglia were more than twice as likely to receive antidepressants compared with those from London or the North West of England, as well as being more likely to report greater life-time histories of self-harm. In an attempt to understand how the differences we found between individuals who had and had not been prescribed SSRIs interacted, we included all the variables where differences were seen within a logistic regression model predicting SSRI prescribing The results suggested that the rationale underlying SSRI prescribing in adolescents differed based on gender; self-harm being the main predicting factor for SSRI prescribing in girls, whilst severity of depressive illness predicted prescribing in boys.

If, as it seems, NICE guidelines, within the UK, are not being systematically followed, the question must therefore be why not? It is possible that issues such as low diagnostic accuracy, a lack of familiarity with current guidelines, insufficiently available adequately trained adolescent mental health practitioner and inadequate psychological services available in secondary and tertiary care [19], may all compromise the chances of such guidance being followed. It is also possible that some psychiatrists disagree with NICE guidelines so chose not to follow them.

It may be argued that in a number of adolescents with severe depression, SSRIs should be the treatment of first choice with psychological therapies added, as remission ensues to aid recovery and prevent relapse [20]. However, the evidence for the effectiveness of SSRIs is derived from studies where they have been prescribed based on depressive symptoms and severity [20, 21]. Little is known regarding their effectiveness when prescribed on the basis of non-depressive hazardous risk behaviour and it is possible that they may be less effective in this population.

Why do the clinical characteristics for prescribing appear to differ between boys and girls? One possibility is that as depression is more common in girls, perhaps, clinicians are wary about prescribing for these symptoms alone and see self-harming behaviour (more common in girls [22, 23]) as a marker of high risk warranting SSRIs. It is possible that as fewer boys present with depression, clinicians feel they require a lower threshold of risk to prescribe.

## Limitations

The main limitation of this study is that we have no clinical measurements available regarding illness severity and quality of life when assessed by the clinicians who prescribed prior to ranodmisation. Therefore, it is theoretically possible that SSRI cases were more severely depressed when first assessed as suffering from MD than their non-SSRI counterparts, when seen before entering the trial. It should be noted that a case-note audit of 80 participating individuals from one of the clinics involved in the study showed that 75 % of the cases had been prescribed medication for less than 3 months prior to randomisation, and more than half of these had received medication for less than 1 month. Although it is possible that there may be some signs of improvement this early on in treatment [24], we believe that this would be unlikely to change the outcome of the study. Indeed, we argue that if this were the case, assessment at the time of prescribing would lead to greater effects in terms of the differences seen. It should be noted that the measurements of risk behaviour and self-harm are based over the lifetime and are, therefore, unlikely to be susceptible to change within this time frame.

We do, however, accept that our study should be considered as correlative rather than indicative.

We believe that the data presented reflects the potential rationale for prescribing SSRIs prior to psychological therapy. When asked prior to randomisation if they had received any previous psychological therapy or counselling, only nine individuals (2 %) reported that they had and only three of these were prescribed SSRIs. However, further questioning by trial therapists suggests that although participants do not appear to have received any evidence-based psychological therapy, a number have had access to certain types of talking therapy including counselling services. This raises the possibility for poor reliability with regard to the recall of previous therapy.

We recognise that some aspects of our findings may be UK specific and our findings would benefit from replication within other international healthcare systems. However, our study uses internationally recognised methods for recruiting adolescents with MDD and therefore, we believe our sample to be comparable with patient groups throughout Europe and worldwide.

It is also possible that there are characteristics of the individuals or their environmental context including family history of mental illness that may account for these findings. Finally, this is a relatively small sample of adolescent patients and is particularly biased towards females, meaning that our findings regarding the prescribing rationale for boys may be associated with a type one or type two error. The participants were also recruited and consented to take part in a randomised controlled trial and therefore, may not be entirely representative of the general population of adolescents with depression. As this may affect the generalizability of our study, these findings require replication in an independent study.

Currently, we conclude that pragmatic SSRI prescribing in the NHS may be based on depressive severity for boys but overt hazardous behaviour for girls. This suggests that there may be greater adherence to NICE guidelines by clinicians treating boys as opposed to girls but this remains to be established. In addition, it appears that prescribing differs in distinct areas of the country. The NICE guidelines have recently been reviewed, however, we believe that before they can be seriously implemented, it is necessary to understand how adolescent MD is currently managed in everyday clinical practice.

## Clinical implications

- The NICE guidance for the treatment of adolescent MD has recently been reviewed, however, to aid our understanding of the optimal management of this debilitating condition, we need to be aware of the reasons clinicians depart from these guidelines. Trial evidence and clinical guidelines should be considered together along with a better understanding of what takes place currently in clinical practice.
- Positive reasons should be recorded in the notes for prescribing SSRIs along with any previous failure of an adequately given psychological treatment. These might explain why clinicians in the UK seem not to be adhering to NICE guidelines.
- Self-harming behaviour should not on its own prompt SSRI prescribing. Instead, prescribing is warranted when suicidality and risk behaviour is combined with moderate to severe depressive symptoms.
- Different reasons may be used for prescribing SSRIs for girls and boys, despite there being no evidence that this is correct practice.

## Electronic supplementary material

Below is the link to the electronic supplementary material.

Supplementary material 1 (DOCX 53 kb)
