# Supplementary material for: Clinical characteristics associated with the prescribing of SSRI medication in adolescents with major unipolar depression
Source: Eur Child Adolesc Psychiatry. 2016 Apr 28;25(12):1287–95. doi: 10.1007/s00787-016-0849-y (PMC5102956; doi:10.1007/s00787-016-0849-y)
Supplement: Supplementary file 1 — Supplementary material 1 (DOCX 53 kb) [file 787_2016_849_MOESM1_ESM.docx]

**Supplementary Material**

‘Clinical characteristics associated with the prescribing of SSRI medication in adolescents with major unipolar depression’

L Cousins, KJ Whitaker, B Widmer, N Midgley, S Byford, B Dubicka, R Kelvin, S Reynolds, C Roberts, F Holland, B Barrett, R Senior, P Wilkinson, M Target, P Fonagy, I Goodyer.

Journal – European Child and Adolescent Psychiatry

Corresponding author – Dr. Lesley Cousins, Department of Psychiatry, University of Cambridge, lesley.cousins@gmail.com

**Supplementary Table 1**

**Multinomial logistic regression analysis investigating the potential effects of research centre on the characteristics of each population**

| C  **Centre** | **Odds ratio** | **S.E.** | **z** | **p** | **95% C.I.** |
| --- | --- | --- | --- | --- | --- |
| **London^a^** | | | | | |
| **Gender** | 0.87 | 0.36 | -0.37 | 0.71 | -0.84 – 0.57 |
| **EQ5D** | 0.65 | 0.57 | –0.73 | 0.47 | -1.54 – 0.71 |
| **HONOSCA** | 1.00 | 0.03 | -0.13 | 0.90 | -0.052 – 0.059 |
| **RTSHIA – Self-harm** | **0.94** | **0.02** | **-3.31** | **0.001** | **-0.086 – -0.022** |
| **Antisocial Behaviour** | 1.02 | 0.05 | 0.32 | 0.75 | -0.08 – 0.12 |
| **MFQ** | 1.02 | 0.02 | 0.85 | 0.40 | -0.020 – 0.050 |
| **Age** | **1.23** | **0.11** | **1.98** | **0.048** | **0.002 – 0.42** |
| **Ethnicity** | **0.13** | **0.35** | **-5.78** | **< 0.001** | **-2.69 – -1.33** |
| **North West^a^** | | | | | |
| **Gender** | 0.98 | 0.29 | -0.087 | 0.93 | -0.59 – 0.54 |
| **EQ5D** | 0.75 | 0.53 | -0.53 | 0.59 | -1.32 – 0.76 |
| **HONOSCA** | **0.94** | **0.02** | **-2.50** | **0.013** | **-0.10 – -0.012** |
| **RTSHIA – Self-Harm** | **0.95** | **0.02** | **-3.62** | **< 0.001** | **-0.082 – -0.024** |
| **Behavioural Checklist** | 1.04 | 0.04 | 0.85 | 0.39 | -0.047 – 0.118 |
| **MFQ** | 1.00 | 0.02 | 0.096 | 0.92 | -0.028 – 0.031 |
| **Age** | 1.10 | 0.09 | 1.02 | 0.31 | -0.087 – 0.28 |
| **Ethnicity** | 0.66 | 0.38 | -1.09 | 0.28 | -1.16 – 0.33 |

^a^ Comparing to East Anglia

**Supplementary Table 2**

**Logistic regression analysis demonstrating the effects of gender on each of the parameters measured with regard to predicting SSRI prescribing.**

|  | **Odds ratio** | **S.E.** | **z** | **p** | **95% C.I.** |
| --- | --- | --- | --- | --- | --- |
| **Male Gender (M)** | 0.14 | 4.23 | -0.47 | 0.64 | -10.26 – 6.33 |
| **EQ5D** | 0.50 | 0.65 | -1.10 | 0.27 | -1.92 – 0.54 |
| **M x EQ5D** | 1.28 | 0.19 | 0.19 | 0.85 | -2.23 – 2.72 |
| **HONOSCA** | 1.05 | 0.03 | 1.57 | 0.12 | -0.01 – 0.11 |
| **M x HONOSCA** | 0.96 | 0.06 | -0.75 | 0.46 | -0.15 – 0.07 |
| **Self-Harm** | **1.03** | **0.02** | **2.00** | **0.045** | **0.001 – 0.065** |
| **M x Self-Harm** | **0.92** | **0.04** | **-2.11** | **0.034** | **-0.16 – -0.006** |
| **Antisocial Behaviour** | 0.89 | 0.06 | -1.78 | 0.076 | -0.24 – 0.01 |
| **M x Antisocial Behaviour** | 0.96 | 0.11 | -0.37 | 0.71 | -0.26 – 0.18 |
| **MFQ** | 0.99 | 0.02 | -0.71 | 0.48 | -0.05 – 0.02 |
| **M x MFQ** | **1.08** | **0.04** | **2.12** | **0.034** | **0.006 – 0.16** |
| **Age** | 1.17 | 0.13 | 1.19 | 0.23 | -0.10 – 0.41 |
| **M x Age** | 1.13 | 0.22 | 0.54 | 0.59 | -0.32 – 0.56 |
| **London** | 0.45 | 0.46 | -1.77 | 0.076 | -1.70 – 0.09 |
| **M x London** | 0.26 | 0.97 | -1.39 | 0.17 | -0.32 – 0.56 |
| **North West** | 0.61 | 0.37 | -1.35 | 0.18 | -1.23 – 0.23 |
| **M x North West** | 0.24 | 0.76 | -1.87 | 0.062 | -2.91 – 0.07 |
| **Ethnicity** | 1.25 | 0.45 | 0.50 | 0.61 | -0.65 – 1.10 |
| **M x Ethnicity** | 0.62 | 0.87 | -0.55 | 0.59 | -2.18 – 1.23 |
